# Improving the teaching skills of residents as tutors/facilitators and addressing the shortage of faculty facilitators for PBL modules

**DOI:** 10.1186/1472-6920-7-34

**Published:** 2007-10-08

**Authors:** Wasim Jafri, Khalid Mumtaz, William P Burdick, Page S Morahan, Rosslynne Freeman, Tabassum Zehra

**Affiliations:** 1 Department of Medicine, The Aga Khan University Hospital, Karachi, Pakistan; 2 FAIMER (Foundation for advancement of medical education) Institutes, Philadelphia, PA, USA

## Abstract

**Background:**

Residents play an important role in teaching of medical undergraduate students. Despite their importance in teaching undergraduates they are not involved in any formal training in teaching and leadership skills. We aimed to compare the teaching skills of residents with faculty in facilitating small group Problem Based Learning (PBL) sessions.

**Methods:**

This quasi experimental descriptive comparative research involved 5 postgraduate year 4 residents and five senior faculty members. The study was conducted with all phase III (Final year) students rotating in Gastroenterology. The residents and faculty members received brief training of one month in facilitation and core principles of adult education. Different aspects of teaching skills of residents and faculty were evaluated by students on a questionnaire (graded on Likert Scale from 1 to 10) assessing i) Knowledge Base-content Learning (KBL), ii) PBL, iii) Student Centered Learning (SCL) and iv) Group Skills (GS).

**Results:**

There were 33 PBL teaching sessions in which 120 evaluation forms were filled; out of these 53% forms were filled for residents and 47% for faculty group. The faculty showed a statistically greater rating in "KBL" (faculty 8.37 Vs resident 7.94; p-value 0.02), "GS" (faculty 8.06 vs. residents 7.68; p-value 0.04). Differences in faculty and resident scores in "the PBL" and "SCL" were not significant. The overall score of faculty facilitators, however, was statistically significant for resident facilitators. (p = .05).

**Conclusion:**

1) Residents are an effective supplement to faculty members for PBL; 2) Additional facilitators for PBL sessions can be identified in an institution by involvement of residents in teacher training workshops.

## Background

There are different ways of learning in the medical school including didactic lectures, class room discussions, small group discussion or tutorials. One of the new learning process is "Problem Based Learning" (PBL). This is an active learning strategy that enables the students to develop critical thinking skills through posing challenges based on clinical case scenarios. PBL also helps students to activate their knowledge and elaborate on hypothesis after critically analyzing it and then resolving problems using all available literature [1]. PBL modules are conducted in our medical school by the faculty members. This is increasingly difficult in view of the increased challenges for the patient care, administrative and bedside teaching activities. Increasing demands in patient care and administrative duties make it increasingly difficult for faculty to set aside necessary time to teach. In order to address this difficulty, the residents in training in the Department of Medicine were selected as extra human resource to help the facilitation of the PBL module. This in turn would promote the necessary teaching and facilitation tool to the residents who are future faculty and also would address to shortage if available faculty for such assignments.

Residents all over the world spend many hours every week teaching medical students and junior residents. Research in medical education has shown that residents (i.e. pre-registration house officers and junior doctors) play an important role in teaching Medical undergraduates [1,2]. Medical students claim that up to a third of their education is derived from residents and feel that each Department should provide minimum guidelines for residents' teaching responsibilities [3]. Residents have important roles as teachers and ward team leaders for the interns and students, yet most residency programs internationally, and none in our country, provide formal training in teaching and leadership skills. Residents felt they would be better teachers if they received some form of training in teaching skills [2,4]. In 1993 a survey in US showed that only 20% of Internal Medicine residency programs featured teaching skills improvement programs, despite the fact that residents provided 62% of inpatient teaching for medical students according to the residency directors, estimates [5,6].

Research has documented that teaching improves the learning of residents [1,7]. Furthermore, upon completion of their Post graduate specialist training, new medical specialists are expected to undertake teaching responsibilities for both medical students and residents in many Medical institutions. It is therefore not surprising that the need has been increasing steadily for a teacher training program for residents as well as the demand for more acknowledgement of the resident's role as a teacher [2,8,9].

We designed a study to compare the senior resident in the Department of Medicine with faculty members as PBL small group facilitators, to address the issue of shortage of faculty facilitators at the Aga Khan University. We also conducted a teaching program to improve the teaching skills of our residents as well as faculty, before they began to facilitate in the PBL modules. This report details the formulation, implementation and evaluation of a pilot study to teach residents to facilitate PBL groups, and to compare the performance of these residents with senior faculty in this task.

## Methods

We conducted a quasi experimental descriptive comparative research. This pilot study involved five residents in postgraduate year 4 (PGY4) and five senior faculty members. The study started in March 2003 and concluded in August 2003. All the residents and faculty members were selected from the same discipline i.e., section of Gastroenterology, Department of Medicine, so that the two groups were similar regarding the topics of teaching sessions. For achieving uniformity, all residents were in their fourth year of training, i.e. having similar work experience. The same standards were used for selection of faculty members as well i.e. they all have an experience of more than 10 years of teaching. The study was approved by the ethics review committee (ERC) of our hospital. A written informed consent was taken from all the participants including the students, residents and faculty members prior to start of this study.

The first step of the study was a meeting with the teaching group comprising senior faculty members and residents, and the Dean of the university. An introduction was given regarding the study, explaining the objectives, methodology and the outcomes anticipated.

## Intervention

The residents and faculty members received introductory training over one month (one 3 hour session each week for a total of 12 hours). There were a total of 12 training hours in teaching skills at the beginning for both groups. We adapted this 12 hours curriculum for teaching skills approach from the successful experience of Morrison et al reported in 2004 [10,11]. These sessions emphasized the learning skills in facilitation and core principles of adult education to faculty and residents. The teaching learning skill sessions included specially designed lectures, (2 hours, 17%), small group teaching (3 and a half hours, 29%), role plays (3 and a half hours, 29%) and critiqued teaching (3 hour-25%). The allocation of time was based on the fact that there is no "one size fits all" approach that can be recommended for either training or evaluation [12]. The main aim of these sessions were to follow the Bringing Education & Service Together (BEST) 8 module which reflected the components of teaching that physicians-in-training [12] needed to learn: 1) leadership/role modeling, 2) orienting learners, 3) giving feedback, 4) bedside teaching, 5) teaching procedures, 6) inpatient teaching, 7) teaching charting, and 8) giving lectures [13,14].

Both residents and senior faculty members received similar structured support for teaching the PBL curriculum. The basic methodology also included explanation of the Kolb Learning Style Inventory [15,16] and the Mezirow Learning Cycle [17].

The study was conducted with all phase III (Final year) students. These students are required to rotate through gastroenterology PBL curriculum for two weeks during their phase III with 5 students present in each rotation. PBL cases on topics in Gastroenterology and Hepatology are employed, with discussion focused on differential diagnosis and management. In addition to the PBL component of the curriculum, each student is on-call under direct supervision of a senior resident and a senior faculty member. During their on-call day, in addition to five hours per week of bedside teaching sessions and small group interactive sessions, students clerk all patients admitted then present and discuss the case with the clinical team. Students attend at least two out patient clinics per week, where they also see patients and discuss their cases with the attending gastroenterologist.

We choose the students for the evaluation because several recent reviews of the literature have concluded that feedback from student evaluations can improve teaching performance. The most popular form of student feedback that is used world wide and that we also adopted is individual standardized ratings where students respond to items describing important dimensions of teaching by marking a scale that has been constructed to reflect successful or unsuccessful performances on those dimensions. Although studies show that feedback from student evaluations improves teacher performance but for how long is yet to be studied. We used students as evaluators to rate the teaching skills of residents and the faculty as they are thought to be the best evaluators of teaching [18].

Tools were built for collection of information on the outcomes. The tools are in the form of evaluation forms on which the students anonymously evaluated and scored the skills of the two facilitator groups. The evaluation forms were developed in a workshop ensuring validity and reliability [19-21] with standard of facilitatory skills and were reviewed by an education expert prior to the start of study with the consensus of all the participants. The group selected the items from pool that were most relevant to the domain of teaching skills in a clinical setting. The tool allowed evaluation in areas identified. The areas of teaching skills were compared with the standards in teaching [18].

These forms were not validated before the study. These forms were also validated during this pilot study. Following aspects of teaching by residents or faculty were evaluated by students on a questionnaire.

• **Knowledge base-content learning**: Probes understanding of material to full extent. Challenges application to other situations. Requires students to relate learning issues to patient's problem.

• **Problem based learning process**: Encourages problem identification and hypothesis generation. Encourages multi-system approach to patient's problem. Helps students identify focused learning issues.

• **Student centered learning**: Active participant in group discussions. Gives students the opportunity to lead a case discussion. Encourages student to interact.

• **Group skills**: Helps resolve group conflict. Models critical listening. Encourages effective presentation of material by student. Helps group to "own" their tutorial and assume responsibility for their individual and collective learning.

• **Facilitator evaluation of students**: Uses learning prescriptions to facilitate setting of unit goals and to evaluate knowledge, skills, attitudes and behavior. Models honest, constructive feedback to individuals and group at the end of each tutorial. Regularly informs students of progress/problems and facilitates follow-up and re-evaluation.

All the aspects listed above were scored on a Likert scale of 1–10 (where 10 is perfect, 9 is exceptional, 8 is excellent, followed by very good, good, acceptable and <5 unsatisfactory).

Evaluation forms were completed by students immediately after each PBL session. At the end of this study, the resident group was also interviewed at the end of this study, regarding their perceived improvement in their teaching skills based on the teaching workshop and experience in PBL module on a scale of 1–5 with 5 being "excellent/very useful.

It was understood that the knowledge of the subject was far better in the faculty then the residents as all of the faculty had at least a decade's experience of teaching and practicing, whereas the residents were comparatively junior and still in the education phase. Therefore no test was done to assess the GI and hepatology knowledge of the residents and the faculty.

The following outcomes were expected from the current study:

1. Short term outcomes:

▪ Evaluation of teaching skills of residents in facilitation of PBL blocks.

▪ Expansion in the number of skilled tutors for facilitation of PBL blocks.

2. Long term outcome:

▪ Induction of residents in PBL curriculum as skilled facilitators for block modules.

▪ Improvement in the teaching skills of residents.

### Statistics

The data was analyzed on the SPSS version 10.0. The students' evaluations of residents and faculty were scored from 1–10 on a Likert scale. The results are presented as mean ± standard deviation for Likert scale. The individual and over all mean score of 5 contributes of learning of residents and faculty were compared using student's t-test. Interview of residents and faculty regarding their perception of these teaching skills sessions were assessed as a dichotomous variable (useful/not useful) and results are presented as Fisher's Exact test. A p-value of < 0.05 was considered significant. A 95% confidence intervals for faculty/residents were calculated and compared using independent sample t-test.

## Results

**Figure 1:**
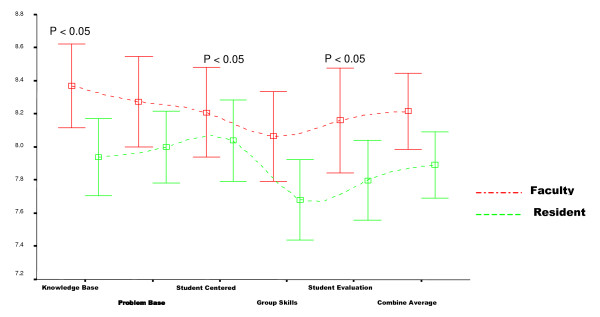
Comparison of Faculty/Resident in different aspects of teaching skills in PBL module showing 95% CI.

There were 33 PBL teaching sessions in which 120 evaluation forms were completed by 53% students in the residents group and 47% in the faculty group. The results for student evaluation forms revealed that the faculty was better facilitators in two of the five teaching domains. The faculty showed a statistically significant rating in "knowledge based learning" (faculty 8.37 Vs resident 7.94; p-value 0.02), "group skills" (faculty 8.06 vs. residents 7.68; p-value 0.04). Differences in faculty and resident facilitators' scores in "the problem based learning process", "student centered learning" and in "students' clinical evaluation" were not statistical significant (p > 0.05). The overall score of faculty facilitators, however, was statistically greater than for resident facilitators. (p = .05) (Table 1). The 95% confidence interval is shown in figure 1.

**Table 1:** Comparison of Residents and Faculty in different aspects of teaching

	**Over all Score**	**Faculty Score**	**Residents Score**	**P-value**
Knowledge Base Learning	8.10 ± 1.13	8.37 ± 1.00	7.94 ± 1.18	0.02
Problem Based Learning	8.10 ± 1.10	8.27 ± 1.07	8.00 ± 1.10	NS
Process Student Centered Learning	8.10 ± 1.18	8.21 ± 1.07	8.04 ± 1.24	NS
Group Skills	7.83 ± 1.18	8.06 ± 1.07	7.68 ± 1.23	0.04
Students Evaluations	7.94 ± 1.24	8.16 ± 1.24	7.80 ± 1.22	NS
Overall average Score	8.02 ± 0.99	8.22 ± 0.91	7.89 ± 1.02	0.04

Results are expressed as mean ± standard deviation.

P-value calculated among Faculty vs Residents.

NS = Not Significant

Four out of five residents found the teaching workshop and participating in the PBL module very useful and improved their teaching skills. One resident, who was relatively junior, asked for further sessions in order to improve his teaching skills. Whereas all of the 5 senior faculty members felt that 12 hr teaching workshop were useful and improved their teaching skills. The residents also agreed that this teaching opportunity has improved their confidence while teaching the students and their overall teaching skills. No difference was ascertained in the responses regarding perception of formal teaching skill training among residents and faculty. [P-value 1.00 (Fisher Exact test)]

## Discussion

Teaching skills courses are taught in a minority of Internal Medicine programs all over the world. Residents do have a more contact hours with the interns and medical students than do faculty on many rotations and are therefore important educators of those learners. Residents as teacher programs can be traced back to the early 1960s [22]. Now a days Programs have become both more specialized and common in the western developed countries, particularly in areas that include Pediatrics, [4,23] Surgery [24], Internal medicine [25], Psychiatry [26], Family medicine [27], etc.

Whilst PBL modules have been conducted by the faculty for many years in our University, prior to this intervention no alternative model had been considered. Overall, there was a better response to the Faculty teaching by the students when compared with the Residents. This confirms our view that there is little substitute for the vast experience faculty members possess.

However, the performance of the residents was also appreciated by the medical students. The scores in three disciplines were not statistically significant when compared with the faculty score, and they have performed very close to the faculty group in three out of five teaching disciplines. Contrary to general belief, our residents taught in a satisfactory manner, which leads us to conclude that the time spent in orientating residents towards teaching skills, and exploring educational frameworks, was useful to them in practice. This suggests that, some teacher training, followed by supported experience, maximize the contribution of residents as teachers of PBL sessions.

Residents in this study themselves rated the teaching workshop and participation in PBL module as being the most helpful dimensions, and valued highly the opportunity to practice teaching skills, delivering teaching to students, interacting with attending physicians and giving constructive feedback.

The experience of teaching the PBL module, together with participation in a teaching workshop improved the teaching performance of residents. Repeating this educational intervention will help Aga Khan University in identifying new facilitators from the resident group, a factor which will contribute to resolving the acute shortage of teachers. This project has encouraged other departments to develop similar interventions, including Cardiology and Pulmonology, which means that more residents will share the experience of being taught to teach, and develop their skills over time.

## Conclusion

In conclusion, we found residents to be an effective supplement to faculty members for facilitation of PBL sessions. With specific education in teaching methods, residents can be helpful in facilitating PBL sessions. Facilitation of the PBL module and participation in teaching workshop also appears to have improved the teaching performance of residents. Involvement of residents in PBL sessions may help institutions identify additional facilitators who can help resolve a shortage issue.

## Competing interests

The author(s) declare that they have no competing interests.

## Authors' contributions

WJ and RF were responsible for the initial design of the study. WJ and KM designed the questionnaire of this study. KM analyzed the data. All authors including WJ, PM, and TZ contributed to editing and writing the manuscript. All authors read and approved the final version of the manuscript.

## Pre-publication history

The pre-publication history for this paper can be accessed here:


